# Epidemiology and Quality of Care for Aboriginal and Torres Strait Islander Peoples Treated by Emergency Medical Services for Stroke in Victoria, Australia: Retrospective Analysis of Linked Data

**DOI:** 10.5694/mja2.70303

**Published:** 2026-09-28

**Authors:** Candice Menezes, Ziad Nehme, Luke J. Burchill, Tegwyn McManamny, Angela Dos Santos, Michelle Crilly, Luke P. Dawson, Benjamin Clissold, David Anderson, Emily Nehme

**Affiliations:** ^1^ Centre for Research and Evaluation Ambulance Victoria Melbourne Victoria Australia; ^2^ School of Public Health and Preventive Medicine Monash University Melbourne Victoria Australia; ^3^ Department of Paramedicine Monash University Melbourne Victoria Australia; ^4^ Department of Medicine University of Melbourne Melbourne Victoria Australia; ^5^ Division of Cardiovascular Diseases Mayo Clinic Rochester New York USA; ^6^ Aboriginal and Torres Strait Islander Leadership Council Australian Stroke Alliance Melbourne Victoria Australia; ^7^ Department of Diversity and Inclusion Ambulance Victoria Melbourne Victoria Australia; ^8^ Department of Cardiology The Alfred Hospital Melbourne Victoria Australia; ^9^ Stroke and Aging Research Group, School of Clinical Sciences Monash University Melbourne Victoria Australia; ^10^ Safer Care Victoria Melbourne Victoria Australia; ^11^ Medical Directorate Ambulance Victoria Melbourne Victoria Australia

**Keywords:** epidemiology, Indigenous health, medical emergency services, stroke

## Abstract

**Objectives:**

To examine the epidemiology of stroke and transient ischaemic attack (TIA), and quality of care provided to Aboriginal and Torres Strait Islander (‘Indigenous’) patients presenting to emergency medical services (EMSs) with stroke or TIA.

**Design:**

Retrospective population‐based data linkage study.

**Setting, Participants:**

Victoria, Australia. Patients aged ≥ 18 years with hospital‐confirmed stroke or TIA who presented to EMSs between 1 January 2015 and 30 June 2019 and were successfully linked with hospital or mortality datasets.

**Main Outcome Measures:**

Thirty‐ and 90‐day mortality, crude and age‐standardised incidence rates, EMS quality of care.

**Results:**

Among 32,163 patients, 287 (0.9%) were Indigenous. Indigenous patients were younger than non‐Indigenous patients (median age, 62 vs. 77 years, *p* < 0.001). Age‐standardised incidence of EMS attendance was higher within the Indigenous population (343 vs. 144 per 100,000 person‐years; incidence rate ratio, 2.383 [95% confidence interval (CI), 2.084–2.711]). Rates of call‐taker identification of stroke or TIA and hospital diagnoses were comparable; however, Indigenous patients were less likely to receive a stroke assessment from paramedics (166/285 [58.2%] vs. 21,271/31,605 [67.3%]; *p* = 0.001) and more frequently taken to stroke‐capable hospitals (257/275 [93.5%] vs. 27,371/30,647 [89.3%]; *p* = 0.027), but less frequently taken to endovascular thrombectomy‐capable hospitals (67/275 [24.4%] vs. 10,015/30,647 [32.7%]; *p* = 0.003). Adjusted mortality was similar between groups (30 days: hazard ratio, 0.814 [95% CI, 0.518–1.279]; 90 days: hazard ratio, 0.805 [95% CI, 0.534–1.214]). However, age‐standardised incidence of 90‐day mortality was higher within the Indigenous population (39 vs. 24 per 100,000 person‐years; incidence rate ratio, 1.621 [95% CI, 1.031–2.398]). Indigenous patients also more often recontacted EMSs for any reason within 90 days (subdistribution hazard ratio, 1.548 [95% CI, 1.194–2.007]).

**Conclusions:**

The age‐standardised incidence of EMS attendance for stroke in the Indigenous population was more than double that for the non‐Indigenous population. Although there was no difference in stroke identification at the time of the emergency call, Indigenous patients were less likely to be evaluated by paramedics for stroke.

## Plain Language Summary

**The Known:** Aboriginal and Torres Strait Islander (‘Indigenous’) peoples have a roughly twofold greater stroke hospitalisation rate than non‐Indigenous patients. However, few studies have investigated variation in pre‐hospital stroke care between Indigenous and non‐Indigenous patients.

**The New:** In Victoria, Australia, the age‐standardised incidence of emergency medical service attendance for stroke within the Indigenous population was more than twice that for the non‐Indigenous population. Rates of stroke identification in emergency calls and stroke and transient ischaemic attack hospital diagnoses were comparable; however, Indigenous patients less frequently received a stroke assessment from paramedics. The age‐standardised incidence of 90‐day mortality was higher for Indigenous Australians.

**The Implications:** Opportunities exist to improve care for Indigenous patients with stroke.

## 1 Introduction

Stroke impacts around 46,000 Australians annually and carries a high burden of mortality and disability [1]. The burden of stroke is not equally distributed across the Australian population, with Aboriginal and Torres Strait Islander (‘Indigenous’) peoples having a hospitalisation rate roughly twofold greater than non‐Indigenous people, and a 2.5‐fold greater mortality rate [2]. In addition, Indigenous patients experience stroke, on average, 16 years earlier than non‐Indigenous Australians [2]. Despite this, little research has investigated whether Indigenous patients receive comparable care to non‐Indigenous patients, or experience disparities in stroke outcomes.

Existing research surrounding stroke care for Indigenous and non‐Indigenous Australians is conflicting, owing to heterogeneity in data definitions and identification of Indigenous status, as well as variation in geographic context. For example, a 2009 national audit suggested Indigenous patients are less likely to receive care according to optimal clinical guidelines or in a stroke unit [3]. Conversely, a New South Wales study (2020) reported similar treatment rates according to Indigenous status for patients admitted to a stroke unit [4]. Literature investigating post‐stroke outcomes and short‐term mortality in Indigenous and non‐Indigenous populations also conflict, with some studies reporting higher mortality in Indigenous populations and others reporting no difference [3, 4, 5, 6]. Importantly, to our knowledge, there is no literature exploring variation in prehospital care.

This study therefore aimed to investigate the epidemiology of stroke and transient ischaemic attack (TIA), and quality of care provided to Indigenous and non‐Indigenous patients with hospital‐confirmed stroke or TIA attended by emergency medical services (EMSs), in Victoria, Australia.

## 2 Methods

### 2.1 Positionality Statement

This study was conceived by an Aboriginal researcher (L.J.B.). The research team included two Aboriginal clinician researchers (A.D.S., M.C.) with expertise in stroke and prehospital care. Aboriginal liaison officers from hospitals represented in the dataset formed an Indigenous advisory group, providing guidance on study design, community needs and priorities. Aboriginal members of the research team (L.J.B., A.D.S., M.C.), which included members of the Kulin Nation from which data were collected, participated across all stages of research, ensuring the priorities identified by Aboriginal liaison officers were centred across the project.

### 2.2 Study Design

This was a retrospective observational data linkage study of adult patients (≥ 18 years old) with confirmed stroke or TIA within Victoria, Australia, between 1 January 2015 and 30 June 2019. We excluded cases of trauma, interhospital transfers and patients with missing Indigenous status. Repeat stroke and TIA presentations within 90 days of the index event were excluded and instead coded as a 90‐day outcome. The inclusive ‘ever‐Indigenous’ approach was taken, whereby patients who identified as Indigenous once within the dataset were classified as Indigenous throughout the entire analysis [7, 8]. This approach ensures that the Indigenous population is not under‐represented [9]. Ethics approval for this Aboriginal and Torres Strait Islander research was obtained from Monash University Human Research Ethics Committee (No. 39764). This study was reported in accordance with the Strengthening the Reporting of Observational Studies in Epidemiology guidelines (Table S1).

### 2.3 Study Setting

Victoria spans 227,500 km^2^ and has a population of 6.7 million inhabitants. Ambulance Victoria is the statewide EMS provider and is two‐tiered, including Mobile Intensive Care Ambulance paramedics and Advanced Life Support paramedics. Emergency Triple Zero (000) calls are triaged using the universal Medical Priority Dispatch System (MPDS), which uses structured questioning to arrive at the patient's primary complaint. Most call types receive an emergency ambulance response. However, predefined low‐acuity call types are referred for secondary triage by an experienced clinician, which may result in emergency ambulance or non‐emergency patient transport service dispatch, referral to an alternative service (including the emergency department [ED]) or provision of self‐care advice.

Upon arrival at the patient, paramedics follow clinical practice guidelines. If paramedics suspect a stroke or TIA, the Melbourne Ambulance Stroke Screen (MASS) is recommended to assess symptoms including facial droop, grip weakness, slurred speech and abnormal blood glucose levels. If stroke symptoms are present, patients are transported to the nearest thrombolysis or endovascular thrombectomy (EVT)‐capable hospital [10]. Within rural and regional areas where stroke‐specific care is limited, 19 hospitals have access to the Victorian Stroke Telemedicine (VST) service, which allows regional and remote hospitals to connect virtually with neurologists in metropolitan areas, providing lower access hospitals with greater capacity to provide care [11].

### 2.4 Data Collection and Sources

A linked dataset was utilised, which included data from: (1) prehospital electronic patient care records; (2) the Victorian Emergency Minimum Dataset, which captures demographic, administrative and clinical data for ED presentations at Victorian public hospitals; (3) the Victorian Admitted Episodes Dataset, which includes information on the causes, effects and nature of illness for all admissions to public and private hospitals; and (4) the Victorian Death Index, which collects information on the date and cause of death. The data linkage process has been described previously [12]. For patients attended by paramedics but not transported to hospital, ED presentations and hospital admissions occurring up to 48 h after calling Triple Zero (000) were linked with the prehospital record. We also obtained population estimates from the Australian Bureau of Statistics to calculate crude and age‐standardised incidence rates.

### 2.5 Definitions

Hospital‐confirmed stroke or TIA was defined as a primary, secondary or tertiary International Classification of Diseases, tenth revision, Australian modification (ICD‐10‐AM) diagnosis code of stroke (I61–I64) or TIA (G459). Sex was defined as the patient's sex at birth. Accessibility/Remoteness Index of Australia scores were used to categorise patient residential and scene addresses based on geographical accessibility to services [13]. Patients in outer regional, remote and very remote areas of Victoria were grouped into one cohort. Socio‐economic status was reported using the Index of Relative Socio‐economic Disadvantage, which groups people and households within an area into deciles based on their relative disadvantage [14]. For analysis, deciles were further collapsed into quintiles. ‘Stroke identified by paramedics’ describes cases where paramedics recorded a final diagnosis of stroke, TIA, subarachnoid haemorrhage or intracranial haemorrhage on their patient care record. ‘Stroke identified by call‐taker’ included cases allocated as Protocol 28 (Stroke) MPDS code during the emergency telephone triage. Pain scores were self‐reported on a scale of 0 (no pain) to 10 (worst imaginable pain). Vital signs were categorised based on Ambulance Victoria's clinical practice guidelines [15]. Receiving hospitals were defined as stroke‐capable if they had a stroke unit and/or the ability to administer thrombolysis, and EVT‐capable if they had the capacity to perform EVT [16]. Subsequent interhospital transfers were not examined, therefore transport time was defined as the duration of transport to the original receiving ED. Days to death, repeat stroke and recontact with the EMS were calculated from the date of the initial emergency call.

### 2.6 Data Analysis

All analyses were performed using Stata 17 statistical software (StataCorp). Crude, age‐specific and age‐standardised incidence and mortality rates per 100,000 person‐years were calculated using 2016 Indigenous and non‐Indigenous population estimates from the Australian Bureau of Statistics, with the 2001 Victorian population used as the reference year [17]. Incidence rates were compared using incidence rate ratios (IRRs) with 95% confidence intervals (CIs). Categorical data are presented as frequencies and proportions and compared using the chi‐squared test. Continuous data are presented as medians and interquartile ranges (IQRs) with comparisons made using the Mann–Whitney *U* test.

Multivariable survival analyses were performed to examine differences in 30‐ and 90‐day mortality, repeat stroke presentation and recontact with the EMS for any reason. For mortality, parametric Weibull models were used based on examination of the baseline hazard function, allowing for a flexible representation of time‐varying risk. Results are presented as hazard ratios (HRs) and 95% CIs. For repeat stroke and recontact, Fine–Gray competing risks regression models were used, with death within 30 or 90 days modelled as a competing risk. Results are presented as subdistribution hazard ratios (sHRs) and 95% CIs. All models were adjusted for Indigenous status, patient age, sex, hospital diagnosis (stroke or TIA), scene remoteness, patient socio‐economic status quintile and comorbidities including previous stroke or TIA, atrial fibrillation, chronic obstructive pulmonary disease and liver disease. Sensitivity analyses were performed in which Indigenous status was classified according to individual hospital admission records, rather than using the ever‐Indigenous approach. For the mortality models, additional sensitivity analyses were performed using multiple imputation with 20 chained equations to account for missingness in the comorbidity and socio‐economic status variables. Rubin's rules were used to pool results. An age‐adjusted logistic regression analysis was also conducted to understand differences in performance of the MASS assessment. The result of this analysis is presented as an adjusted odds ratio with 95% CI. *p* values of < 0.05 were considered statistically significant.

## 3 Results

After exclusions, 32,163 confirmed stroke or TIA patients were included (Figure 1). Of these patients, 287 (0.9%) identified as Indigenous. Of the 31,876 non‐Indigenous patients, 27,142 (85.1%) had a hospital diagnosis of stroke and 4734 (14.9%) had a hospital diagnosis of TIA. Among Indigenous patients, 246 (85.7%) were diagnosed with stroke, and 41 (14.3%) with TIA.

**FIGURE 1:**
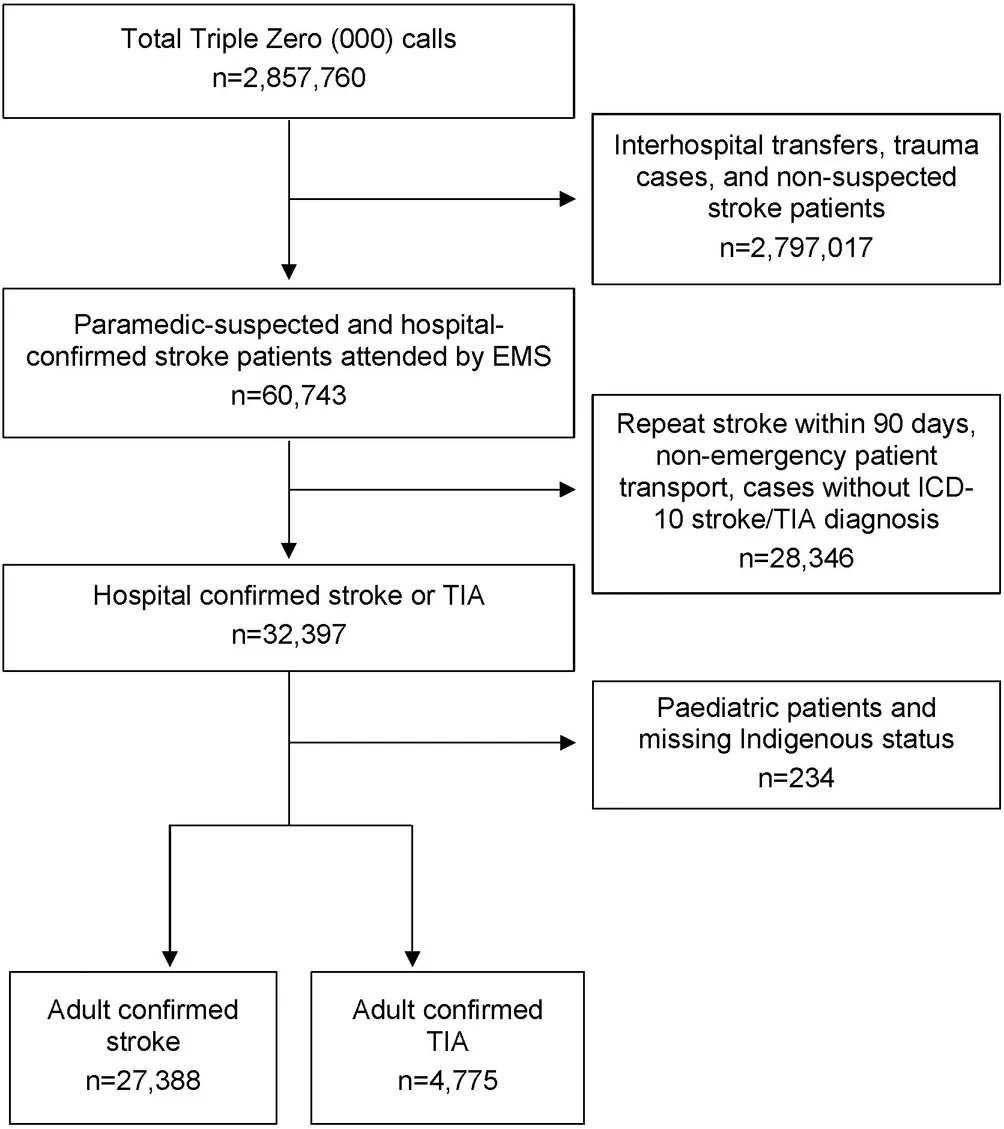
Patient selection. EMS, emergency medical services, ICD, international classification of diseases; TIA, transient ischaemic attack.

### 3.1 Incidence

The crude incidence of EMS attendance for stroke was higher in the Indigenous population than the non‐Indigenous population (220 vs. 164 per 100,000 person‐years; IRR, 1.346 [95% CI, 1.194–1.512], Figure 2A, Table S2). After age standardisation, incidence in the Indigenous population was more than twice that in the non‐Indigenous population (343 vs. 144 per 100,000 person‐years; IRR, 2.383 [95% CI, 2.084–2.711]). Age‐specific incidence across 10‐year age categories is presented in Figure 2B and was consistently higher within the Indigenous population.

**FIGURE 2:**
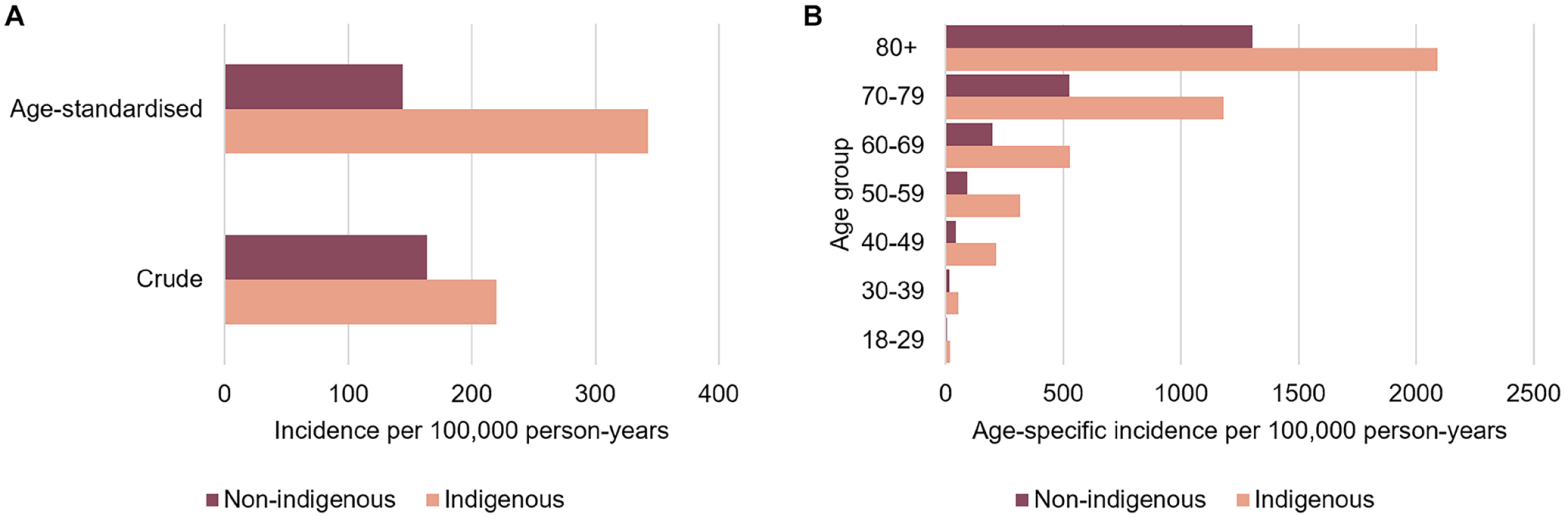
Crude, age‐standardised and age‐specific incidence per 100,000 person‐years within Indigenous and non‐Indigenous populations.

### 3.2 Baseline Characteristics

Table 1 shows the baseline characteristics of the study population. Indigenous patients were younger (median age, 62 years [IQR, 49–72 years] vs. 77 years [IQR, 66–85 years]; *p* < 0.001) and more often resided in areas of lower socioeconomic status. Most patients resided in metropolitan areas, but Indigenous patients had a higher proportion of residents in inner regional, outer regional and remote areas. Indigenous patients had an increased history of liver disease and more frequently presented with a rapid heart rate. Non‐Indigenous patients had an increased history of atrial fibrillation.

**TABLE 1:** Baseline characteristics of Indigenous and non‐Indigenous patients.

	Overall	Non‐Indigenous	Indigenous	*p*
Total	32,163	31,876	287	
Age, median (IQR)	77 (66–85)	77 (66–85)	62 (49–72)	< 0.001
Females, *n* (%)^a^	15,893 (49.9)	15,743 (49.9)	150 (52.8)	0.328
Missing	Not provided	329 (1.0%)	< 5	—
Patient residential location, *n* (%)				
Metropolitan^a^	23,005 (73.5)	22,851 (73.6)	154 (55.8)	< 0.001
Inner regional^a^	6776 (21.6)	6684 (21.5)	92 (33.3)	< 0.001
Outer regional/remote^a^	1527 (4.9)	1497 (4.8)	30 (10.9)	< 0.001
Missing	855 (2.7)	844 (2.6)	11 (3.8)	—
Socio‐economic status, *n* (%)				
Quintile 1 (lowest)^a^	6899 (24.0)	6797 (23.9)	102 (40.5)	< 0.001
Quintile 2^a^	5893 (20.5)	5838 (20.5)	55 (21.8)	0.612
Quintile 3^a^	5815 (20.3)	5771 (20.3)	44 (17.5)	0.265
Quintile 4^a^	5626 (19.6)	5594 (19.7)	32 (12.7)	0.006
Quintile 5 (highest)^a^	4455 (15.5)	4436 (15.6)	19 (7.5)	< 0.001
Missing	3475 (10.8)	3440 (10.8)	35 (12.2)	—
Patient event location, *n* (%)				
Metropolitan^a^	23,555 (73.8)	23,398 (74.0)	157 (55.5)	< 0.001
Inner regional^a^	6830 (21.4)	6736 (21.3)	94 (33.2)	< 0.001
Outer regional/remote^a^	1521 (4.8)	1489 (4.7)	32 (11.3)	< 0.001
Missing	Not provided	253 (0.8)	< 5	—
Pre‐existing comorbidities, *n* (%)				
Stroke/TIA^a^	9315 (30.7)	9219 (30.6)	96 (36.1)	0.054
Myocardial infarction^a^	4538 (14.9)	4497 (14.9)	41 (15.4)	0.826
Renal diseases^a^	1073 (3.5)	1064 (3.5)	9 (3.4)	0.895
COPD^a^	2135 (7.0)	2106 (7.0)	29 (10.9)	0.013
Liver disease^a^	192 (0.6)	184 (0.6)	8 (3.0)	< 0.001
Diabetes^a^	6839 (22.5)	6772 (22.5)	67 (25.2)	0.294
Dementia^a^	2400 (7.9)	2385 (7.9)	15 (5.6)	0.170
Atrial fibrillation^a^	4867 (16.0)	4853 (16.1)	14 (5.3)	< 0.001
Missing	1781 (5.5)	1760 (5.5)	21 (7.3)	—
Initial EMS vital signs, *n* (%)				
Impaired conscious state (GCS score < 15)^a^	12,828 (40.9)	12,717 (40.9)	111 (39.8)	0.701
Missing	806 (2.5)	798 (2.5)	8 (2.8)	—
Fever (≥ 38°C)^a^	722 (2.4)	714 (2.4)	8 (3.0)	0.534
Missing	2038 (6.3)	2020 (6.3)	18 (6.3)	—
Any pain^a^	4972 (16.4)	4917 (16.4)	55 (20.3)	0.084
Missing	1895 (5.9)	1879 (5.9)	16 (5.6)	—
Normal oxygen saturation (≥ 92%)^a^	28,274 (93.6)	28,032 (93.6)	242 (90.6)	0.051
Missing	1940 (6.0)	1920 (6.0)	20 (7.0)	—
Normal blood glucose^a^	7986 (28.0)	7908 (28.0)	78 (31.0)	0.296
Missing	3652 (11.4)	3617 (11.3)	35 (12.2)	—
Normal systolic blood pressure (≥ 100 mmHg)^a^	30,436 (97.2)	30,170 (97.2)	266 (95.3)	0.062
Missing	845 (2.6)	837 (2.6)	8 (2.8)	—
Respiratory rate (breaths/min)				
12–16^a^	17,449 (55.7%)	17,308 (55.7%)	141 (50.5%)	0.084
17–20^a^	10,411 (33.2%)	10,308 (33.2%)	103 (36.9%)	0.186
Missing	813 (2.5%)	805 (2.5%)	8 (2.8%)	—
Heart rate (beats/min)				
Normal (60–100)^a^	25,472 (81.3%)	25,264 (81.3%)	208 (74.6%)	0.004
Moderately rapid (100–120)^a^	3195 (10.2%)	3151 (10.1%)	44 (15.8%)	0.002
Irregular (> 120 or < 60)^a^	2681 (8.6%)	2654 (8.5%)	27 (9.7%)	0.500
Missing	815 (2.5%)	807 (2.5%)	8 (2.8%)	—

Abbreviations: COPD, chronic obstructive pulmonary disease; EMS, emergency medical service; GCS, Glasgow Coma Scale; TIA, transient ischaemic attack.

^a^ Presented as a proportion of patients for whom data were not missing.

### 3.3 Prehospital Care Quality

Prehospital care is presented in Table 2. Identification of stroke within the emergency call was similar between groups. However, Indigenous patients were significantly less likely to receive a MASS assessment from paramedics (166/285 [58.2%] vs. 21,271/31,605 [67.3%]; *p* = 0.001), even after adjusting for age (adjusted odds ratio, 0.721 [95% CI, 0.569–0.915]). This included less frequent assessment for hand grip weakness, facial droop and speech deficits. However, among assessed patients, there were no differences in the rate of deficits identified. Indigenous patients were also significantly less likely to receive an electrocardiogram. Across the state, Indigenous patients were more frequently taken to stroke‐capable hospitals; however, fewer were taken to EVT‐capable hospitals. Among patients attended in metropolitan Melbourne, there was no difference in the rate of transport to stroke‐capable hospitals (138/150 [92.0%] vs. 19,938/22,586 [88.3%]; *p* = 0.157) or EVT‐capable hospitals (64/150 [42.7%] vs. 9559/22,586 [42.3%]; *p* = 0.932). No differences were identified in ambulance response times or time spent on scene, although transport time to hospital was shorter for Indigenous patients.

**TABLE 2:** Prehospital care of Indigenous and non‐Indigenous patients.

	Overall	Non‐Indigenous	Indigenous	*p*
Total	32,163	31,876	287	
Call taker identified stroke, *n* (%)	16,107 (50.1)	15,965 (50.1)	142 (49.5)	0.838
Paramedic intervention, *n* (%)				
MASS assessment completed	21,437 (67.2)	21,271 (67.3)	166 (58.2)	0.001
Speech assessed^a^	26,040 (81.7)	25,834 (81.7)	206 (72.3)	< 0.001
Speech deficit^b^	11,961 (45.9)	11,858 (45.9)	103 (50.0)	0.240
Hand grips assessed^a^	26,839 (84.2)	26,615 (84.2)	224 (78.6)	0.010
Hand grips deficit^b^	12,163 (45.3)	12,053 (45.3)	110 (49.1)	0.253
Facial droop assessed^a^	24,150 (75.7)	23,954 (75.8)	196 (68.8)	0.006
Facial droop deficit^b^	9450 (39.1)	9375 (39.1)	75 (38.3)	0.803
Blood glucose taken^a^	28,511 (89.4)	28,259 (89.4)	252 (88.4)	0.588
Missing	Not provided	271 (0.9)	< 5	—
Electrocardiogram taken^a^	29,237 (91.7)	28,987 (91.7)	250 (87.7)	0.015
Missing	Not provided	271 (0.9%)	< 5	—
Any paramedic intervention^a^	16,746 (53.3)	16,590 (53.3)	156 (55.9)	0.388
Intubation^a^	Not provided	473 (1.5)	< 5	0.906
Intravenous access^a^	11,032 (35.1)	10,939 (35.2)	93 (33.3)	0.524
Analgesia				
Analgesia given^a^	4773 (15.2)	4724 (15.2)	49 (17.6)	0.271
Analgesia refused^a^	418 (1.3)	409 (1.3)	9 (3.2)	0.006
Missing interventions, *n* (%)	774 (2.4)	766 (2.4)	8 (2.8)	—
Stroke or TIA identified by paramedics, *n* (%)^a^	17,334 (55.6)	17,193 (55.6)	141 (50.9)	0.114
Missing, *n* (%)	990 (3.1)	980 (3.1)	10 (3.5)	—
Time metrics				
000 cal to ambulance arrival at patient (min), median (IQR)	11.5 (8.5–17.3)	11.5 (8.5–17.3)	10.8 (8.3–18.2)	0.485
Missing, *n* (%)	872 (2.7%)	863 (2.7%)	9 (3.1%)	—
Time on scene (min), median (IQR)	18 (13–25)	18 (13–25)	18 (13–25)	0.527
Missing, *n* (%)	683 (2.1)	676 (2.1)	7 (2.4)	—
Transport duration (min), median (IQR)^c^	19 (12–28)	19 (12–28)	16 (9–25)	< 0.001
Transported to hospital, *n* (%)^a^	31,313 (99.0)	31,034 (99.0)	279 (99.3)	0.617
Missing, *n* (%)	531 (1.7)	525 (1.6)	6 (2.1)	—
Lights and sirens transport to hospital, *n* (%)^c^	14,394 (46.0)	14,267 (46.0)	127 (45.5)	0.880
Type of hospital, *n* (%)^c^				
Stroke‐capable hospital	27,628 (89.4)	27,371 (89.3)	257 (93.5)	0.027
EVT‐capable hospital	10,082 (32.6)	10,015 (32.7)	67 (24.4)	0.003
Missing	Not provided	387 (1.2)	< 5	—

Abbreviations: EVT, endovascular thrombectomy; IQR, interquartile range; MASS, Melbourne Ambulance Stroke Screen; TIA, transient ischaemic attack.

^a^ Presented as a proportion of patients for whom data were not missing.

^b^ Presented as a proportion of assessed patients.

^c^ Presented for transported patients for whom data were not missing.

### 3.4 In‐Hospital Care Quality

Hospital care is presented in Table 3. Indigenous patients were more likely to leave against medical advice after both ED presentation and hospital admission, and less likely to be admitted to hospital, but more likely to be admitted to an intensive care unit (ICU) during admission. Unadjusted in‐hospital mortality was higher in non‐Indigenous patients (12/248 [4.8%] vs. 2965/28,830 [10.3%]; *p* = 0.005).

**TABLE 3:** Hospital care for Indigenous and non‐Indigenous patients.

	Overall	Non‐Indigenous	Indigenous	p
Total	32,163	31,876	287	
Admitted to ED, *n* (%)	30,538 (94.9)	30,254 (94.9)	284 (99.0)	0.002
ED triage category, *n* (%)^a^				
Resuscitation	1833 (6.0)	1811 (6.0)	22 (7.7)	0.214
Emergency	16,102 (52.7)	15,954 (52.7)	148 (52.1)	0.835
Urgent	9790 (32.1)	9700 (32.1)	90 (31.7)	0.894
Ambulance offload < 40 min, *n* (%)^a^	25,003 (85.3)	24,770 (85.3)	233 (86.3)	0.739
Missing, *n* (%)	1214 (4.0)	1201 (4.0)	14 (4.9)	—
ED length of stay (min), median (IQR)^b^	302 (199–516)	302 (199–516)	311 (202.5–488.5)	0.875
Discharged within 4 h, *n* (%)^a^	12,023 (39.4)	11,912 (39.4)	111 (39.1)	0.921
Discharge direction after ED, *n* (%)^a^				
Ward	28,086 (92.0)	27,829 (92.0)	257 (90.5)	0.357
Home	1413 (4.6)	1399 (4.6)	14 (4.9)	0.807
Left against advice	114 (0.4)	106 (0.4)	8 (2.8)	< 0.001
Aged care facility	Not provided	92 (0.3)	< 5	0.884
Died in ED	Not provided	220 (0.7)	< 5	0.458
Other	Not provided	608 (2.0)	< 5	0.253
Admitted to hospital, *n* (%)	29,078 (90.4)	28,830 (90.4)	248 (86.4)	0.021
Admitted to ICU, *n* (%)^c^	2627 (9.0)	2590 (9.0)	37 (14.9)	0.001
ICU length of stay (h), median (IQR)	44 (22–92.5)	44 (22–92)	47 (28.5–132)	0.467
Admission discharge direction, *n* (%)^c^				
Transfer to other facility	10,009 (34.4)	9930 (34.4)	79 (31.9)	0.393
Discharge to private accommodation/home	14,422 (49.6)	14,289 (49.6)	133 (53.6)	0.202
Discharge to aged care facility	1494 (5.1)	1482 (5.1)	12 (4.8)	0.830
Left against medical advice	176 (0.6)	164 (0.6)	12 (4.8)	< 0.001
Death	2977 (10.2)	2965 (10.3)	12 (4.8)	0.005
Hospital admission length of stay (days), median (IQR)	3 (1–7)	3 (1–7)	3 (1–6)	0.617

Abbreviations: ED, emergency department; ICU, intensive care unit; IQR, interquartile range.

^a^ Presented as a proportion of patients admitted to the ED for whom data were not missing.

^b^ For patients admitted to the ED.

^c^ Presented as a proportion of patients admitted to hospital.

### 3.5 Patient Outcomes

Age‐standardised incidence of 90‐day mortality was higher within the Indigenous than the non‐Indigenous population (39 vs. 24 per 100,000 person‐years; IRR, 1.621 [95% CI, 1.031–2.398], Figure S1). Unadjusted and adjusted patient outcomes are presented in Table 4. Indigenous patients had lower unadjusted mortality at 30 and 90 days. However, after adjustment, there was no difference in mortality (30 days: HR, 0.814 [95% CI, 0.518–1.279]; 90 days: HR, 0.805 [95% CI, 0.534–1.214]). Instead, age, pre‐existing conditions and socioeconomic status were associated with mortality outcomes (Table S3). Indigenous patients more often recontacted the EMS for any reason within 90 days (sHR, 1.548 [95% CI, 1.194–2.007]).

**TABLE 4:** Outcomes of Indigenous and non‐Indigenous patients.

	Overall	Unadjusted comparisons			Adjusted HR/sHR (95% CI)
		Non‐Indigenous	Indigenous	*p*	
Total	32,163	31,876	287		
Mortality, *n* (%)					
Within 48 h of 000 call	1536 (4.8)	1528 (4.8)	8 (2.8)	0.113	—
30 days	4644 (14.4)	4620 (14.5)	24 (8.4)	0.003	0.814 (0.518–1.279)
90 days	5671 (17.6)	5643 (17.7)	28 (9.8)	< 0.001	0.805 (0.534–1.214)
Repeat stroke, *n* (%)					
30 days	522 (1.6)	517 (1.6)	5 (1.7)	0.872	0.900 (0.333–2.428)
90 days	927 (2.9)	918 (2.9)	9 (3.1)	0.796	0.909 (0.427–1.937)
Recontact for any reason, *n* (%)					
30 days	2612 (8.1)	2575 (8.1)	37 (12.9)	0.003	1.214 (0.816–1.807)
90 days	5532 (17.2)	5455 (17.1)	77 (26.8)	< 0.001	1.548 (1.194–2.007)

Abbreviations: CI, confidence interval; HR, hazard ratio; sHR, subdistribution hazard ratio.

### 3.6 Sensitivity Analyses

Sensitivity analyses in which Indigenous status was classified according to individual hospital admission records corroborate the findings of our primary analysis, except for the rate of hospital admission and adjusted 90‐day recontact, which no longer differed significantly between Indigenous and non‐Indigenous patients (Tables S4–S6). Sensitivity analyses using multiple imputation to account for missing data supported the primary mortality findings (Table S7).

## 4 Discussion

Our investigation into the epidemiology of stroke and TIA, and quality of care provided to Indigenous and non‐Indigenous patients with stroke or TIA attended by EMSs in Victoria has several key findings. Age‐standardised incidence within the Indigenous population was more than twice that in the non‐Indigenous population, and Indigenous patients were a median of 15 years younger than non‐Indigenous patients. Rates of stroke identification in the emergency call and stroke or TIA hospital diagnoses were comparable, demonstrating strengths within several components of the care pathway. However, Indigenous patients less frequently received a stroke assessment from paramedics, were more likely to leave hospital against medical advice and were more likely to recontact the EMS for any reason within 90 days. Although there was no difference in adjusted mortality, the age‐standardised incidence of 90‐day mortality was higher for Indigenous Australians.

Our findings suggest that improving paramedics' completion of stroke assessments (MASS) should be a priority in prehospital care. It is not clear from our findings why Indigenous patients were less likely to receive a stroke assessment or electrocardiogram. One explanation may be that Indigenous patients were younger (median age, 62 years), highlighting the need for stroke assessment pathways to be responsive to populations where stroke may occur earlier than expected. Data from the Stroke Foundation suggest that only 25% of stroke patients in Australia are under the age of 65 years [1]. Importantly, our age‐adjusted analysis also demonstrated reduced odds of receiving a MASS assessment among Indigenous patients, suggesting that factors beyond age, including differences in presentation and health system processes, are likely to account for this disparity.

Another explanation may be that Indigenous populations have previously reported higher rates of haemorrhagic strokes [2, 18]. Symptom presentation of haemorrhagic stroke, including headaches, vomiting and facial weakness, overlaps with the presentation of other conditions, including migraines, seizures, hypoglycaemia and Bell palsy [19]. As such, stroke symptoms may not always be recognised through standard assessment approaches. These findings suggest that there remains a potential need to increase EMS education surrounding the recognition of non‐hallmark stroke symptoms, and to ensure that stroke assessment tools are used equitably across different populations.

However, clinician and/or system‐level bias may have also affected Indigenous patients, as has previously been reported in emergency settings [20] and stroke care [21]. Systemic inequities in prehospital stroke care have been demonstrated in the United States, where white patients were more likely to be evaluated by a stroke team than African American patients [22]. Similarly, Hispanic and Asian patients were less likely than non‐Hispanic white patients to be correctly identified as having a stroke by EMSs [23]. Together, these findings highlight the need for stroke services to better recognise how individual and process biases may affect stroke outcomes, especially for Indigenous Australians who may present younger and atypically [1].

A higher proportion of Indigenous patients were taken to stroke‐capable hospitals. This finding likely reflects the higher proportion of Indigenous patients living in regional and remote areas, where most major public hospitals have access to the VST service. Importantly, transport to a stroke‐capable facility does not imply that thrombolysis was administered, and additional investigation is warranted. The increased proportion of Indigenous patients residing in regional and remote areas also explains the reduced likelihood of their transport to EVT‐capable hospitals. Notably, no such disparity was observed among patients attended within metropolitan Melbourne. Currently, only five EVT‐capable hospitals exist in Victoria, and all are in metropolitan Melbourne. For metropolitan patients, paramedics may have favoured transport to EVT‐capable hospitals, even if this involved bypassing smaller facilities and an extended transport duration.

In hospital, a higher proportion of Indigenous patients were admitted to ICUs. Regionality may also explain this finding, with ICU admission being the closest alternative to EVT‐capable centres in regional areas. Our study also suggests that Indigenous patients were more likely to leave against medical advice. This finding has been reported consistently across Australian health services and is increasingly understood as reflecting health system factors rather than individual patient characteristics alone. Previous studies suggest that experiences of cultural unsafety, racism, communication barriers, competing family and community responsibilities, and limited responsiveness of services to Indigenous needs may contribute to discharge decisions [24]. Viewed through this lens, the increased hazard of EMS recontact within 90 days may reflect ongoing unmet healthcare needs and opportunities to strengthen continuity of culturally safe stroke care following hospital discharge.

There was no significant difference in adjusted mortality at 30 or 90 days; however, age‐standardised incidence of 90‐day mortality per 100,000 population was higher in the Indigenous population than in the non‐Indigenous population. Until now, age‐standardised mortality has not been studied in an Indigenous cohort within Victoria, although rates in a Western Australian, South Australian and Northern Territory cohort were reported to be around twofold higher in Indigenous patients [2]. Previous studies investigating the impact of age on patient recovery and mortality have suggested that younger patients have a higher chance of optimal recovery, lower stroke recurrence rates and lower mortality rates than older patients [25]. Despite being substantially younger at stroke onset, Indigenous patients did not experience the survival advantage typically observed in younger stroke populations. This finding highlights the enduring influence of broader social, structural and healthcare determinants on outcomes.

Understanding how stroke services respond to Indigenous peoples' health needs is an important step towards strengthening culturally safe, responsive and accountable systems of care. Our findings identify opportunities to improve care across the stroke continuum, while also highlighting strengths within existing pathways upon which future improvements can be built. Consistent with contemporary Indigenous health frameworks, these improvements should be developed through genuine partnerships with Indigenous communities, organisations and leaders, recognising Indigenous peoples as experts in the design, delivery and evaluation of services that affect their health [5, 26]. Strengthening Indigenous leadership, cultural safety and accountability within mainstream stroke services has the potential to support Indigenous concepts of health and wellbeing, while improving experiences and outcomes of care.

### 4.1 Limitations

Our study has some limitations. Data on previously reported confounders such as stroke severity, disability scores [25, 26] and new placement in a nursing home upon hospital discharge were not available, and neither were data on stroke subtypes and ED management. This study was based on hospital‐confirmed stroke cases attended by a single EMS, and the linkage rate with hospital data for patients known to be transported by EMSs to public hospitals has been reported to be 83.3% [12]. Individuals with stroke who did not engage EMSs, whether due to cultural, geographical or other barriers to access, are not accounted for in incidence calculations. As such, the true incidence of stroke in our population is likely to be under‐reported.

### 4.2 Conclusion

The age‐standardised incidence of EMS attendance to patients with stroke in the Indigenous population was twice that for the non‐Indigenous population, and Indigenous patients were a median of 15 years younger at stroke onset. Although rates of stroke identification by call‐takers were comparable, Indigenous patients were less likely to receive a stroke assessment by paramedics. Systemic barriers to care quality may exist for Indigenous patients and opportunities exist to improve care across the stroke continuum.

## Author Contributions

**Candice Menezes:** formal analysis and writing – original draft. **Ziad Nehme:** supervision, methodology, and writing – review and editing. **Luke J. Burchill:** conceptualization, and writing – review and editing. **Tegwyn McManamny:** writing – review and editing. **Angela Dos Santos:** writing – review and editing. **Michelle Crilly:** writing – review and editing. **Luke P. Dawson:** writing – review and editing. **Benjamin Clissold:** writing – review and editing. **David Anderson:** writing – review and editing. **Emily Nehme:** data curation, project administration, supervision, methodology, and writing – review and editing.

## Funding

Ziad Nehme is supported by a Future Leader Fellowship from the National Heart Foundation of Australia (no. 105690) and an Emerging Leadership Investigator Grant from the National Health and Medical Research Council (no. 2034615).

## Conflicts of Interest

The authors declare no conflicts of interest.

## Supporting information


**Table S1:** STROBE statement—checklist of items that should be included in reports of observational studies.
**Table S2:** Crude, age‐standardised and age‐specific incidence of EMS attendance for stroke among Indigenous and non‐Indigenous populations, per 100,000 person‐years.
**Figure S1:** Crude and age‐standardised incidence of 90‐day mortality following EMS attendance for stroke, per 100,000 person‐years, within Indigenous and non‐Indigenous populations.
**Table S3:** Multivariable analyses of 30‐ and 90‐day mortality.
**Table S4:** Baseline characteristics of Indigenous and non‐Indigenous patients, with Indigenous status classified according to individual hospital admission records.
**Table S5:** Pre‐hospital and hospital care of Indigenous and non‐Indigenous patients, with Indigenous status classified according to individual hospital admission records.
**Table S6:** Patient outcomes, with Indigenous status classified according to individual hospital admission records.
**Table S7:** Adjusted mortality outcomes at 30 and 90 days, using multiple imputation to account for missingness in comorbidities and socio‐economic status.

## Data Availability

Data are available upon reasonable request from the corresponding author and will be subject to ethics committee and data custodian approval.
