# Supplementary material for: Epidemiology and Quality of Care for Aboriginal and Torres Strait Islander Peoples Treated by Emergency Medical Services for Stroke in Victoria, Australia: Retrospective Analysis of Linked Data
Source: Med J Aust. 2026 Sep 28;224(10):e70303. doi: 10.5694/mja2.70303 (PMC13620277; doi:10.5694/mja2.70303)
Supplement: Supplementary file 1 [file MJA2-224-0-s001.pdf]

Supporting Information

Supplementary material

This appendix was part of the submitted manuscript and has been peer reviewed.
It is posted as supplied by the authors.

Appendix to: C. Menezes, Z. Nehme, L. Burchill, et al. Epidemiology and Quality of Care for Aboriginal and Torres Strait Islander Peoples Treated by Emergency Medical Services for Stroke in Victoria, Australia: Retrospective Analysis of Linked Data. *Medical Journal of Australia* 2026; doi: 10.5694/mja2.70303.

Table S1. STROBE Statement—checklist of items that should be included in reports of observational studies

Page numbers refer to the submitted manuscript, not the published article

	Item No	Recommendation	Page No
**Title and abstract**	1	(*a*) Indicate the study's design with a commonly used term in the title or the abstract	1
		(*b*) Provide in the abstract an informative and balanced summary of what was done and what was found	2
**Introduction**			
Background/rationale	2	Explain the scientific background and rationale for the investigation being reported	4
Objectives	3	State specific objectives, including any prespecified hypotheses	4
**Methods**			
Study design	4	Present key elements of study design early in the paper	5
Setting	5	Describe the setting, locations, and relevant dates, including periods of recruitment, exposure, follow-up, and data collection	5
Participants	6	(*a*) *Cohort study*—Give the eligibility criteria, and the sources and methods of selection of participants. Describe methods of follow-up<br>*Case-control study*—Give the eligibility criteria, and the sources and methods of case ascertainment and control selection. Give the rationale for the choice of cases and controls<br>*Cross-sectional study*—Give the eligibility criteria, and the sources and methods of selection of participants	5
		(*b*) *Cohort study*—For matched studies, give matching criteria and number of exposed and unexposed<br>*Case-control study*—For matched studies, give matching criteria and the number of controls per case	
Variables	7	Clearly define all outcomes, exposures, predictors, potential confounders, and effect modifiers. Give diagnostic criteria, if applicable	5,7
Data sources/ measurement	8*	For each variable of interest, give sources of data and details of methods of assessment (measurement). Describe comparability of assessment methods if there is more than one group	6-8

Bias	9	Describe any efforts to address potential sources of bias	
Study size	10	Explain how the study size was arrived at	5
Quantitative variables	11	Explain how quantitative variables were handled in the analyses. If applicable, describe which groupings were chosen and why	6-8
Statistical methods	12	(*a*) Describe all statistical methods, including those used to control for confounding	7-8
		(*b*) Describe any methods used to examine subgroups and interactions	7-8
		(*c*) Explain how missing data were addressed	5,8
		(*d*) *Cohort study*—If applicable, explain how loss to follow-up was addressed<br>*Case-control study*—If applicable, explain how matching of cases and controls was addressed<br>*Cross-sectional study*—If applicable, describe analytical methods taking account of sampling strategy	
		(*e*) Describe any sensitivity analyses	
**Results**			
Participants	13*	(a) Report numbers of individuals at each stage of study—eg numbers potentially eligible, examined for eligibility, confirmed eligible, included in the study, completing follow-up, and analysed	9
		(b) Give reasons for non-participation at each stage	N/A
		(c) Consider use of a flow diagram	Fig1
Descriptive data	14*	(a) Give characteristics of study participants (eg demographic, clinical, social) and information on exposures and potential confounders	9-10<br>Tab1
		(b) Indicate number of participants with missing data for each variable of interest	Tables
		(c) *Cohort study*—Summarise follow-up time (eg, average and total amount)	8
Outcome data	15*	*Cohort study*—Report numbers of outcome events or summary measures over time	*10-11*
		Case-control study—Report numbers in each exposure category, or summary measures of exposure	
		Cross-sectional study—Report numbers of outcome events or summary measures	
Main results	16	(*a*) Give unadjusted estimates and, if applicable, confounder-adjusted estimates and their precision (eg, 95% confidence interval). Make clear which	9-11

		confounders were adjusted for and why they were included	
		(*b*) Report category boundaries when continuous variables were categorized	8-11
		(*c*) If relevant, consider translating estimates of relative risk into absolute risk for a meaningful time period	
Other analyses	17	Report other analyses done—eg analyses of subgroups and interactions, and sensitivity analyses	7-8
**Discussion**			
Key results	18	Summarise key results with reference to study objectives	12
Limitations	19	Discuss limitations of the study, taking into account sources of potential bias or imprecision. Discuss both direction and magnitude of any potential bias	15-16
Interpretation	20	Give a cautious overall interpretation of results considering objectives, limitations, multiplicity of analyses, results from similar studies, and other relevant evidence	16
Generalisability	21	Discuss the generalisability (external validity) of the study results	12-16
**Other information**			
Funding	22	Give the source of funding and the role of the funders for the present study and, if applicable, for the original study on which the present article is based	17

*Give information separately for cases and controls in case-control studies and, if applicable, for exposed and unexposed groups in cohort and cross-sectional studies.

Note: An Explanation and Elaboration article discusses each checklist item and gives methodological background and published examples of transparent reporting. The STROBE checklist is best used in conjunction with this article (freely available on the Web sites of PLoS Medicine at http://www.plosmedicine.org/, Annals of Internal Medicine at http://www.annals.org/, and Epidemiology at http://www.epidem.com/). Information on the STROBE Initiative is available at www.strobe-statement.org.

Table S2. Crude, age-standardised, and age-specific incidence of EMS attendance for stroke among Indigenous and non-Indigenous populations, per 100,000 person-years

	Indigenous	Non-Indigenous	IRR [95% CI]
Crude	220	164	1.346 [1.194,1.512]
Age-standardised	343	144	2.383 [2.084,2.711]
Age-specific			
18-29	18	5	3.602 [1.535,7.227]
30-39	53	16	3.394 [1.796,5.852]
40-49	213	44	4.832 [3.592,6.371]
50-59	315	92	3.415 [2.599,4.409]
60-69	528	198	2.662 [2.042,3.412]
70-79	1182	523	2.257 [1.684,2.963]
80+	2088	1302	1.603 [1.139,2.193]

IRR: incidence rate ratio; CI: confidence interval

Figure S1. Crude and age-standardised incidence of 90-day mortality following EMS attendance for stroke, per 100,000 person-years, within Indigenous and non-Indigenous populations.

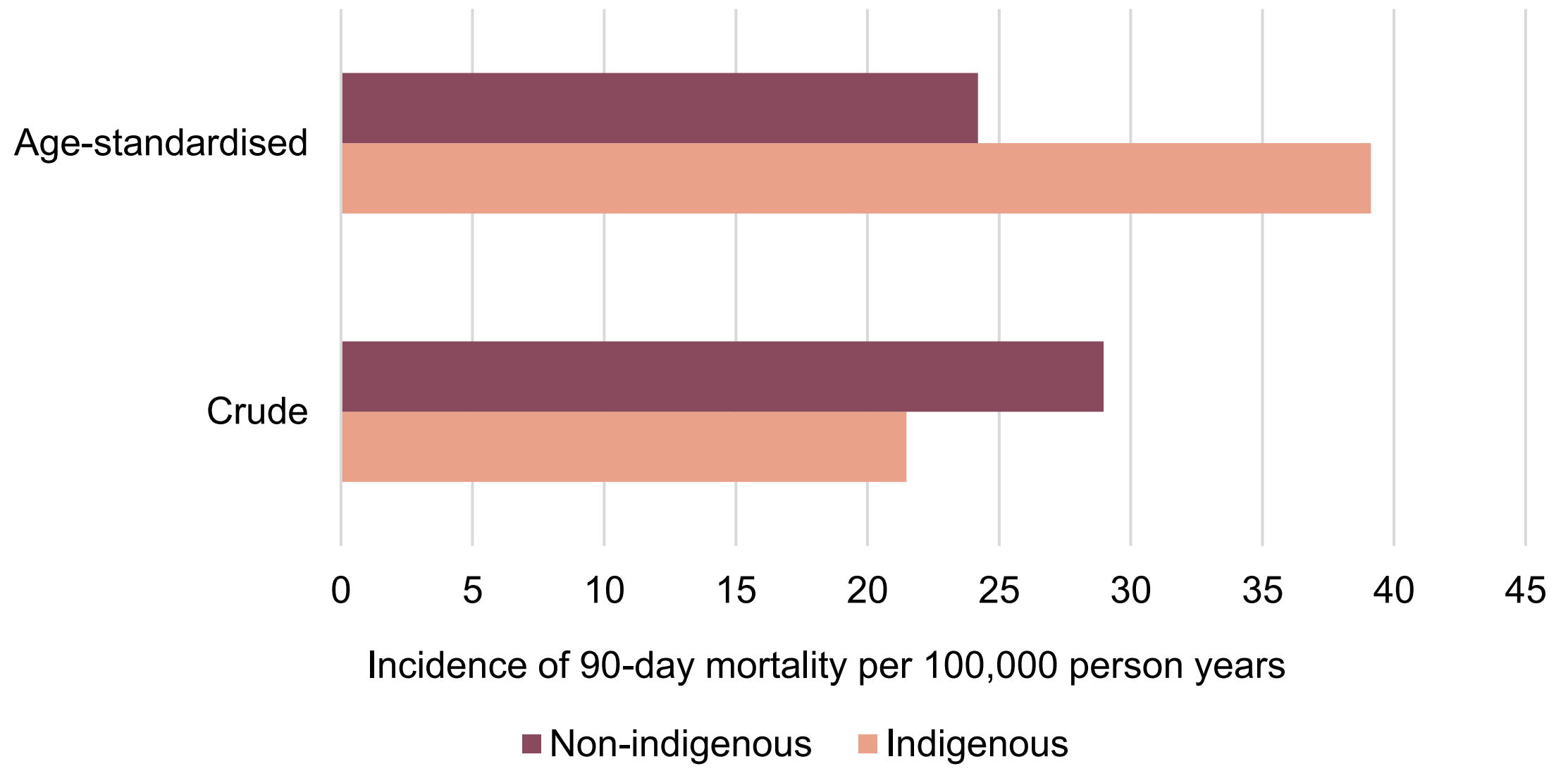

Table S3. Multivariable analyses of 30- and 90-day mortality

	30-day mortality	90-day mortality
	HR [95% CI]	HR [95% CI]
Indigenous status	0.814 [0.518, 1.279]	0.805 [0.534, 1.214]
Age, per year	1.044 [1.041, 1.047]	1.047 [1.044, 1.050]
Male gender	0.877 [0.823, 0.934]	0.896 [0.847, 0.949]
TIA hospital diagnosis (relative to stroke)	0.112 [0.088, 0.142]	0.173 [0.145, 0.206]
Pre-existing conditions		

Stroke / TIA	0.827 [0.772, 0.886]	0.842 [0.791, 0.896]
COPD	1.230 [1.102, 1.372]	1.278 [1.159, 1.410]
Liver disease	2.626 [1.955, 3.528]	2.660 [2.026, 3.491]
Atrial fibrillation	1.262 [1.172, 1.360]	1.229 [1.148, 1.315]
Event location		
Metropolitan	Ref	Ref
Regional	1.063 [0.984, 1.149]	1.062 [0.990, 1.139]
Remote / very remote	1.366 [1.191, 1.567]	1.358 [1.199, 1.538]
Socioeconomic status		
1 (lowest)	Ref	Ref
2	0.899 [0.820, 0.987]	0.874 [0.804, 0.951]
3	0.915 [0.834, 1.004]	0.905 [0.832, 0.984]
4	0.918 [0.835, 1.009]	0.909 [0.834, 0.990]
5 (highest)	0.884 [0.798, 0.980]	0.869 [0.791, 0.953]

TIA: transient ischaemic attack; HR: hazard ratio; CI: confidence interval; COPD: chronic obstructive pulmonary disease

Table S4. Baseline characteristics of Indigenous and non-Indigenous patients, with Indigenous status classified according to individual hospital admission records

	Non-Indigenous	Indigenous	p-value
Total	31934	229	
Age, median (IQR)	77 (66-85)	61 (48-72)	<0.001
Females, n (%)	15774 (49.9)	119 (52.7)	0.411
Missing	329 (1.0)	<5	-
Patient residential location, n(%)			
Metropolitan	22889 (73.6)	116 (53.2)	<0.001
Inner regional	6697 (21.5)	79 (36.2)	<0.001
Outer regional/remote	1504 (4.8)	23 (10.6)	<0.001
Missing	844 (2.6)	11 (4.8)	-
Socioeconomic status, n(%)			
Quintile 1 (lowest)	6817 (23.9)	82 (40.8)	<0.001
Quintile 2	5846 (20.5)	47 (23.4)	0.317
Quintile 3	5778 (20.3)	37 (18.4)	0.510
Quintile 4	5600 (19.7)	26 (12.9)	0.017
Quintile 5 (highest)	4446 (15.6)	9 (4.5)	<0.001
Missing	3,447 (10.8)	28 (12.2)	-
Patient event location, n(%)			
Metropolitan	23437 (74.0)	118 (52.4)	<0.001
Inner regional	6750 (21.3)	80 (35.6)	<0.001
Outer regional/remote	1494 (4.7)	27 (12.0)	<0.001
Missing	253 (0.8)	<5	-
Pre-existing comorbidities, n(%)			
Stroke / TIA	9245 (30.6)	70 (33.5)	0.373

Myocardial Infarction	4508 (14.9)	30 (14.4)	0.813
Renal diseases	1065 (3.5)	8 (3.8)	0.816
Chronic obstructive pulmonary disease	2113 (7.0)	22 (10.5)	0.047
Liver disease	185 (0.6)	7 (3.4)	<0.001
Diabetes	6788 (22.5)	51 (24.4)	0.511
Dementia	2385 (7.9)	15 (7.2)	0.698
Atrial Fibrillation	4857 (16.0)	10 (4.8)	<0.001
Missing	1761 (5.5)	20 (8.7)	-
Initial EMS Vital Signs, n(%)			
Impaired conscious state (GCS<15)	12736 (40.9)	92 (41.4)	0.871
Missing	799 (2.5)	7 (3.1)	-
Fever (≥38°C)	714 (2.4)	8 (3.7)	0.198
Missing	2,023 (6.3)	15 (6.6)	-
Any pain	4932 (16.4)	40 (18.6)	0.387
Missing	1,881 (5.9)	14 (6.1)	-
Normal oxygen saturation (≥92%)	28080 (93.6)	194 (91.1)	0.141
Missing	1924 (6.0)	16 (7.0)	-
Normal blood glucose	7928 (28.0)	58 (29.0)	0.754
Missing	3623 (11.4)	29 (12.7)	-
Normal systolic blood pressure (≥100 mmHg)	30225 (97.2)	211 (95.1)	0.053
Missing	838 (2.6)	7 (3.1)	-
Respiratory rate (breaths/min), n (%)			
12-16	17336 (55.7)	113 (50.9)	0.152
17-20	10332 (33.2)	79 (35.6)	0.451
Missing	806 (2.5)	7 (3.1)	-
Heart rate (beats/min), n (%)			
Normal (60-100)	25307 (81.3)	165 (74.3)	0.008
Moderately rapid (100-120)	3162 (10.2)	33 (14.9)	0.021
Irregular (>120 or <60)	2657 (8.5)	24 (10.8)	0.227
Missing	808 (2.5)	7 (3.1)	-

TIA: Transient Ischemic Attack; EMS: Emergency Medical Services; GCS: Glasgow Coma Scale.

Table S5. Pre-hospital and hospital care of Indigenous and non-Indigenous patients, with Indigenous status classified according to individual hospital admission records

	**Non-Indigenous**	**Indigenous**	**p-value**
Total	31934	229	
Call taker identified stroke, n (%)	15995 (50.1)	112 (48.9)	0.722
MASS assessment completed, n (%)	21309 (67.3)	128 (56.4)	<0.001
Speech assessed	25880 (81.7)	160 (70.5)	<0.001
Hand grips assessed	26662 (84.2)	177 (78.0)	0.010
Facial droop assessed	24000 (75.8)	150 (66.1)	0.001
Blood glucose taken	28311 (89.4)	200 (88.1)	0.524
Missing	271 (0.9)	<5	
ECG taken, n (%)	29038 (91.7)	199 (87.7)	0.028
Missing	271 (0.9)	<5	
Stroke/TIA identified by paramedics, n (%)	17222 (55.6)	112 (50.9)	0.159
Time Metrics (mins), median (IQR)			
000 call to ambulance arrival at patient	11.5 (8.5-17.3)	11.9 (8.4-18.4)	0.795
Missing	864 (2.7)	8 (3.5)	-
*Transport duration**	19 (12-28)	15 (8-28)	<0.001
Transported to hospital, n (%)	31091 (99.0)	222 (99.6)	0.401
Missing	525 (1.6)	6 (2.6)	
Type of hospital*, n (%)			
Stroke capable hospital	27421 (89.3)	207 (94.5)	0.013
EVT capable hospital	10031 (32.7)	51 (23.3)	0.003
Missing	288 (1.3)	<5	-
Admitted to ED, n (%)	30,311 (94.9)	227 (99.1)	0.004
Discharge direction after ED†, n (%)			
Ward	27875 (92.0)	211 (93.0)	0.585
Home	1403 (4.6)	10 (4.4)	0.873
Left against advice	111 (0.4)	<5	0.019
Aged care facility	92 (0.3)	<5	0.709
Died in ED	220 (0.7)	<5	0.613
Other	610 (2.0)	<5	0.092
Admitted to hospital, n (%)	28876 (90.4)	202 (88.2)	0.257
Admitted to ICU^, n (%)	2594 (9.0)	33 (16.3)	<0.001
Admission discharge direction^, n (%)			
Transfer to other facility	9944 (34.4)	65 (32.2)	0.501
Discharge to private accommodation/home	14315 (49.6)	107 (53.0)	0.336
Discharge to aged care facility	1485 (5.1)	9 (4.5)	0.659
Left against medical advice	165 (0.6)	11 (5.5)	<0.001
Death	2967 (10.3)	10 (5.0)	0.013

*Presented for transported patients. †Presented as a proportion of patients admitted to ED. ^Presented as a proportion of patients admitted to hospital.
MASS: Melbourne Ambulance Stroke Screen; ECG: electrocardiogram; TIA: transient ischaemic attack; EVT: endovascular thrombectomy; ED: emergency department; ICU: intensive care unit.

Table S6. Patient outcomes, with Indigenous status classified according to individual hospital admission records

	Unadjusted			Adjusted
	Non-Indigenous	Indigenous	p-value	HR / sHR [95% CI]
Total	31934	229		
Mortality, n (%)				
Within 48 hours of 000 call	1529 (4.8)	7 (3.1)	0.221	-
30 days	4622 (14.5)	22 (9.6)	0.037	0.931 [0.577, 1.502]
90 days	5645 (17.7)	26 (11.4)	0.012	0.946 [0.615, 1.454]
Repeat stroke, n (%)				
30 days	520 (1.6)	<5	0.368	0.286 [0.040, 2.025]
90 days	922 (2.9)	5 (2.2)	0.526	0.664 [0.247, 1.787]
Recontact for any reason, n (%)				
30 days	2584 (8.1)	28 (12.2)	0.022	1.043 [0.646, 1.682]
90 days	5477 (17.2)	55 (24.0)	0.006	1.354 [0.994, 1.845]

HR: hazard ratio; sHR: subdistribution hazard ratio; CI: confidence interval.

Table S7. Adjusted mortality outcomes at 30 and 90 days, using multiple imputation to account for missingness in comorbidities and socioeconomic status

	HR [95% CI]
30 days	0.832 [0.551, 1.254]
90 days	0.803 [0.550, 1.174]
